# Global Assessment of Seasonal Potential Distribution of Mediterranean Fruit Fly, *Ceratitis capitata* (Diptera: Tephritidae)

**DOI:** 10.1371/journal.pone.0111582

**Published:** 2014-11-06

**Authors:** Anna M. Szyniszewska, Andrew J. Tatem

**Affiliations:** 1 Department of Geography, University of Florida, Gainesville, Florida, United States of America; 2 Department of Geography and Environment, University of Southampton, Highfield, Southampton, United Kingdom; 3 Fogarty International Center, National Institutes of Health, Bethesda, Maryland, United States of America; International Atomic Energy Agency, Austria

## Abstract

The Mediterranean fruit fly (Medfly) is one of the world's most economically damaging pests. It displays highly seasonal population dynamics, and the environmental conditions suitable for its abundance are not constant throughout the year in most places. An extensive literature search was performed to obtain the most comprehensive data on the historical and contemporary spatio-temporal occurrence of the pest globally. The database constructed contained 2328 unique geo-located entries on Medfly detection sites from 43 countries and nearly 500 unique localities, as well as information on hosts, life stages and capture method. Of these, 125 localities had information on the month when Medfly was recorded and these data were complemented by additional material found in comprehensive databases available online. Records from 1980 until present were used for medfly environmental niche modeling. Maximum Entropy Algorithm (MaxEnt) and a set of seasonally varying environmental covariates were used to predict the fundamental niche of the Medfly on a global scale. Three seasonal maps were also produced: January-April, May-August and September-December. Models performed significantly better than random achieving high accuracy scores, indicating a good discrimination of suitable versus unsuitable areas for the presence of the species.

## Introduction

Many invasive alien species are directly associated with biodiversity loss, ecosystem service changes, and negative impacts on human health, agriculture, forestry and fisheries. In Europe alone, these losses and impacts are estimated to cost at least EUR 12 billion per year [1]. The Mediterranean fruit fly, *Ceratitis capitata* (Wiedemann), commonly referred to as Medfly, is considered one of the world's most destructive pests [2]. It is a highly polyphagus species, able to feed on over 300 hosts and known to be capable of adapting to a wide range of climates [2]–[4]. It causes significant damage to fruits and vegetables, and its economic impacts are substantial.

Medfly originated from sub-Saharan Africa and in early 19^th^ century was identified in southern parts of Europe, from where it subsequently spread to other parts of the globe [5]. It is currently present in Mediterranean Europe and the Middle East, in most parts of Africa including Indian Ocean islands, South and Central America, western Australia and the Pacific region. It is a quarantine pest and countries with established Medfly populations have significant trade barriers imposed to their exports. The pest has been established for about a century in Hawaii and despite persistent and costly eradication efforts, is repeatedly detected into Florida and California [6], [7]. It is estimated that the cost of each of its previous incursions into the US (eradication and industry loss) ranged from US$300,000 to US$200 m [8]. Medfly outbreaks in California during the past 25 years have cost taxpayers nearly $500 million, while the Medfly outbreak in Florida's Tampa Bay region in 1997 resulted in $25 million spent on eradication [9], which is significantly less than the cost of potential establishment. It has been estimated that the cost of controlling established Medfly in the State of California alone could range between $493 million to $875 million, and imposition of trade embargo from Asian countries would result in additional revenue losses of $564 million and cost more than 14,000 jobs [10]. The eastern Mediterranean region also experienced substantial losses linked to fruit fly infestations estimated at US$192 m [11].

Comprehensive global information on Medfly occurrence, both in spatial and temporal terms, is crucial for understanding not only the current and historical extent of its occurrence, but also the conditions where it is able to survive and areas susceptible to potential invasion and establishment. For similar reasons, it is also essential to track historical spread routes and the history of invasion. Occurrence records with temporal reference are important for understanding the drivers of Medfly seasonal population dynamics, which can be valuable for guiding eradication and control strategies. Commonwealth Agricultural Bureaux International (CABI), European Plant Protection Organization (EPPO) and International Atomic Energy Agency (IAEA) are examples of institutions maintaining records on where Medfly is established [12]–[15]. These sources define Medfly presence status at the country scale and less frequently, on the provincial scale. Their quality, according to the most current spatial and temporal information, varies and there are no widely-available expert opinion maps defining the environmental range of known Medfly occurrence.

Recent years have seen a handful of studies aiming to define the potential distribution of Medfly. In a study by De Meyer *et al.* [16], a genetic algorithm for rule-set prediction (GARP) and principal component analysis (PCA) were used to estimate the potential geographical range of Medfly using native range distributional data derived from a database maintained by the Royal Museum for Central Africa. This data was complemented by non-native range information gathered from the literature and electronic resources. Outputs showed areas of high and low suitability for Medfly presence globally without providing information on what constituted the threshold for such categories, or any seasonal changes. CLIMEX (http://www.csiro.au/solutions/ps1h3) was used in a different study to assess the seasonal and year-to-year variation in climatic suitability for Medfly worldwide with emphasis on Argentina and Australia [17]. No occurrence data were used for the modeling, but rather parameters of its population dynamics were used, specifically a CLIMEX growth index derived from a study of Medfly populations in Thessaloniki [18]. Gutierrez and Ponti [19] also assessed the invasive potential of Medfly in California and Arizona using GRASS-GIS, based on age-structured dynamics of Medfly life stages and temperature variability in the region. MaxEnt outperformed GARP in a study [20] which aimed to assess the potential distribution of three fruit fly species including Medfly in China. A set of environmental variables describing temperature and precipitation, as well as worldwide occurrence records were used in the modelling.

It is well documented in regional studies from several areas of the world that Medfly has a highly seasonal pattern to its population dynamics [3], [21]–[29]. However, spatiotemporal datasets to quantify these patterns on a global scale have yet to be assembled, while previous global mapping of the suitability for Medfly presence has not accounted for seasonal shifts. Here we present the results of a study that has focused on constructing the most comprehensive database on confirmed Medfly occurrence records, the timing of these records and their locations. Moreover, information on hosts, life stages and capture method were also recorded. Finally, seasonally-varying gridded environmental variables including temperature, precipitation, elevation and normalized vegetation index (NDVI) were linked to these records in a niche modeling framework to produce predictions of the annual and seasonal distributions of suitability for Medfly presence on a global scale, with an aim of identifying regions that can be potential risk areas for Medfly invasions depending on the season.

## Materials and Methods

### Occurrence data

Confirmed detection location data for *C. capitata* were searched for in online open-access museum collections data, published articles, reports and conference proceedings. The literature search resulted in 158 publications and reports containing potential data to be reviewed [2]–[5], [7], . Of these publications, 101 contained information about Medfly detection that could be geolocated, and 64 contained information about the month when Medfly was observed. A database was constructed to store historical data pertaining to Medfly detection locations. For each entry, information about the author, year and type of publication, country, two administrative levels and locality, georeferenced location and source of coordinates, the quality of information about the location, year and month of the occurrence record, sampling technique, developmental stage of Medfly and host plant were recorded. This data record protocol was built upon that pioneered for recent studies of malaria vectors worldwide [162]–[165]. Locations of Medfly observations were georeferenced, either based on coordinates included in the source material or dependent on the name of the location found in the source, and the geolocation source, method and accurracy is identified in the database. Additional supporting sources of information on the Medfly occurrence locations were obtained from the Global Biodiversity Information Facility (GBIF http://data.gbif.org), Belgian Biodiversity Platform (BeBIF www.biodiversity.be) and the Royal Museum of Central Africa (http://projects.bebif.be/enbi/fruitfly) [16].

### Covariates used in modeling

A suite of environmental variables was constructed in preparation for use in niche modeling in consideration of the pest's environmental limiting factors (Table 1 and 2). Medfly has four stages of development: (1) female adults deposit eggs under the skin of susceptible fruits or vegetables, where (2) eggs hatch to produce larvae. (3) Larvae feed on the pulp of the host before complete the larval development and abandon the fruit to the soil, where (4) larvae pupate and after methamorphosis adults emerge from the soil. The length of time required to complete the Medfly lifecycle in tropical summer weather conditions is in the range of 21 to 30 days [166]. In cool climates it can take well over 200 days [18], [134]. Its presence is also influenced by the availability of hosts. In tropical areas, due to overlapping host phenology, Medfly populations are able to persist year-round or during the majority of the year, whereas in temperate regions, the host-present period is considerably shorter. Medfly, as with other insects, is known to be sensitive to climate, and apart of host availability, one of the main limitations to its development is low temperature that may hinder its ability to overwinter [18], [29], plus high precipitation which may have an adverse impact on the pupae development in the soil [3], [24]. Thermal requirements of insect species are often derived in laboratory conditions and vary with developmental stage, environmental conditions and their geographic origin [167], [168]. The optimum temperature threshold for Medfly development is estimated to be between 21°C and 26.7°C, and below 10°C and above 35°C the development stops [140], [169], [170]. The ability to overwinter at high altitudes on the fringes of suitable areas is very limited and dependent on the availability of hosts favoring slow growth, inside which pest could survive [21], [82]. Given these factors, we obtained land surface temperature (LST) and normalized difference vegetation index (NDVI) images from the Advanced Very High Resolution Radiometer (AVHRR) satellite sensor. The products are available at 8×8 km spatial resolution for over a 20-year time series and downloadable via the Goddard Flight Center's Web Site (http://daac.gsfc.nasa.gov/). Digital elevation model(DEM) data were obtained from the Shuttle Radar Topography Mission (SRTM) [http://www2.jpl.nasa.gov/srtm/]. Finally, annual and quarterly average, minimum and maximum precipitation data were derived from the Worldclim database (http://www.worldclim.org/). The data represent interpolated rainfall measures derived from the world-wide network of weather stations for the time period of 1950–2000 [171].

**Table 1:** Data sets used in the development of ecological niche models, including source and spatial resolution.

Variables in the annual model	Source	Spatial resolution
Minimum Land Surface Temperatures (LST)	AVHRR	5 km
Maximum LST	AVHRR	5 km
Elevation	SRTM	5 km
Minimum precipitation	WorldClim	5 km
Maximum precipitation	WorldClim	5 km
Mean Normalized Difference Vegetation Index (NDVI)	AVHRR	5 km

**Table 2:** The list of the data sets used in the development of seasonal ecological niche models including source and the spatial resolution. Seasonal model 1: Jan–Apr, 2; May–Aug and 3: Sep–Dec.

Variables in the seasonal model	Source	Spatial resolution	Seasonal model
Average LST in the season	AVHRR	5 km	2, 3
Average LST of the coldest month in the season	AVHRR	5 km	1, 3
Average LST of the warmest month in the season	AVHRR	5 km	1
Elevation	SRTM	5 km	1, 2, 3
Precipitation total of the wettest month	WorldClim	5 km	1, 2, 3
Precipitation total of the driest month	WorldClim	5 km	1, 2, 3
Average quarterly NDVI in the season	AVHRR	5 km	1, 2, 3

All of the gridded datasets underwent a number of processing steps prior to being used in the modelling [162], [165], [172]. The size, location and extent have been matched for every layer. For datasets where the remotely sensed information was multi-temporal, Fourier analysis was used to ordinate the data by decomposing the temporal signal into an additive series of harmonics of different seasonal frequencies [162]–[165], [172]. All of the gridded datasets were resampled to produce matching extents and a grid cell size of 5 km×5 km. The layers used in the modelling here do not include locations above 59°N, as they were not available for all datasets.

All of the data layers were tested for pairwise Pearson correlation prior to building and running the model. Although MaxEnt is known to be a stable model in the face of correlated variables [173], those with the high correlation coefficients (r> = 0.85) were excluded from the analysis. These were average precipitation and LST in the general model, and average precipitation in the seasonal model. Average LST was removed from the first season, average LST of the coldest month was removed from the second season and the average LST of the warmest month remained as a covariate only in the first season model.

### Choice of seasons

The year was divided into three Medfly-relevant seasons for seasonal mapping to strike a balance between ensuring that the seasonal variation in Medfly occurrence was captured, and having sufficient datapoints to produce reliable maps for each time period. Dividing year according to calendar seasons did not correspond well with the activity of Medflies, especially in the northern hemisphere, where the seasonal differences tend to be most pronounced due to a larger proportion of land located in higher latitudes. On the northern limits of the Medfly distribution, the pest tends to be inactive between January and April. It is unable to overwinter in egg or adult stage, but there is evidence of an ability to survive low temperatures in fruit hosts as a larvae and to a significantly lesser degree, in the pupal stage [18]. The onset of pest activity starts anywhere between May and August, depending on location and condition in a particular year. The peak of Medfly activity is observed in the fall – usually between September and November, and a sharp decline is observed between November and December. This led us to divide the year into three seasons (January–April, May–August and September–December), which is both significant from the phenological point of view for Medfly, and at the same time preserves some common environmental characteristics of the seasons.

### Maximum entropy modelling

The Maximum Entropy Modelling tool (MaxEnt version 3.3.3 k) was used to map the potential distribution of Medfly. The model estimates species' potential distributions by finding the maximum entropy distribution, in other words, distribution closest to uniform [174], [175]. In the model, the environmental values found at the detection localities impose certain constraints on the output distribution. The constraints are expressed as simple functions of the environmental variables called features, and each feature in the model should have a mean close to the empirical average. The model looks for a set of probability distributions that satisfy the constraints and chooses the most unconstrained one [175]. In the logistic output of the model, every grid square has an assigned value between 0 and 100, which represents the relative suitability of species occurrence.

There were many reasons that dictated the choice of this model. Most importantly, in a review of 16 species modeling methods, MaxEnt was among the best performing methods when evaluated using the area under the curve (AUC) and correlation statistics [174]. Secondly, the method holds a strict mathematical definition and can accommodate diverse types of predictor variables - both categorical and continuous. Moreover, it does not require absence data, can handle a relatively low sample size and gives a simple to interpret continuous output. Finally, the method is well documented and available for free download (http://www.cs.princeton.edu/~schapire/maxent).

The default MaxEnt model parameters have been calibrated on a wide range of data (a convergence threshold of 10^−5^, a maximum iteration value of 500 and the maximum number of background points as 10000). These settings are recommended to achieve good model performance for species at ecological equilibrium [176]. Because Medfly is an invasive organism, we modified these settings to better predict the nature of the potential niche of an invader. The convergence threshold was left at the default of 10^−5^ and the number of iterations was increased to 5000 to allow the model adequate time for convergence and avoid under- or over-prediction of the relationships. We explored the choice of features and increased model regularization. MaxEnt allows various feature types to be used by default (if there are enough sample points on species presence available), which results in complex functions. Using less or only one feature is recommended for simpler models and we chose hinge features which allow the model to fit nonlinear functions of varying complexity, but without the sudden steps of threshold features [173]. Increased regularization parameters increase the degree of level smoothing, however the AUC score of our model was consequently declining with increased regularization. Therefore, we left the regularization setting at the default of 1. The output maps illustrate the mean results of the replicated runs. There are no precise scientific guidelines that dictate the choice of settings, so our choice was based on a visual assessment of their influence on the partial dependence plots, AUC scores and the prediction maps.

The logistic habitat-suitability output values were used for the model output which is simple to interpret (probability range of occurrence between 0 and 1). The model estimates the relative influence of each variable used in the prediction. It is scaled so the sum of the relative influence of each variable adds to 100, with higher numbers indicating stronger contribution on the outcome [177]. In the case where multiple Medfly occurrence points in our constructed database were registered at a single location, only one record was used in the MaxEnt modelling. Only points of occurrence and sources from 1980 onwards were taken into consideration to build the models.

By default, MaxEnt selects its own background samples from the entire study region, which implies that this entire space is available to species and surveillance [178]. Another option is to include a mask that will allow MaxEnt to choose a background sample only from pre-selected areas. In the case of Medfly these could be areas that are accessible for Medfly – where the species currently is present and no eradication efforts are currently ongoing, and no quarantine measures against Medfly are in place. It could also include areas that were accessible to Medfly over decades. Finally, the mask could help represent the sampling bias of Medfly occurrence records, however this was not feasible here as the data comes from various sources over long temporal scales. Since the goal of this study was to represent Medfly fundamental and not realized niche, and also how environmental variables favorable for its presence are changing according to the season, plus we only have political boundaries of Medfly current presence and not expert drawn distribution maps, we did not use a mask for the general potential niche model, but we applied a mask for the three seasonal models. The mask was obtained by using a probablility threshold of Medfly occurrence equal to or above 0.1 from the general model. It helped to avoid overprediction of the model and filter out areas that can only be suitable for Medfly occurrence during a few months of the year and therefore cannot sustain populations over climatically unfavorable months.

### Model testing

The accuracy of the distribution models was evaluated by partitioning the data within MaxEnt into training (75%) and testing (25%) subsets and performing validation statistical analyses on each of the partitions. Each of the settings were run on 30 replicates using a subsample run type with a random seed, so that Maxent could average the results from all of the models created. Firstly, the area under the Receiver Operating Characteristic (ROC) was used to measure model performance. The plot of the ROC curve is illustrative of the ratio of correctly classified positives to the total number of positive cases (sensitivity) versus the false positive rate (specificity) at all thresholds of presence-absence classification. In this case, we do not have actual absence data in the study. Therefore, tests show whether the model classifies presence more accurately than a random prediction. The ROC plot for a model whose predictive ability is the equivalent of random assignment will lie near the diagonal, where the true positive rate equals the false positive rate for all thresholds. AUC is therefore a good measure of the overall model performance and has a possible range of 0–1, where 0 indicates that prediction is equal to a random assignment while an AUC score of 1 indicates a perfect presence-absence prediction. Secondly, a threshold-dependent binomial test of omission was performed. If in a specific cell we observe a value of 0.10 or above, that cell is classified as suitable for Medfly. This approach transforms the prediction output from continuous into binary. The number of Medfly suitable cells was compared to the number of cells known to have had Medfly presence. A one tailed binomial test was used to find out whether the model outperformed a random model predicting Medfly to be present in the same number of cells. MaxEnt provides test statistics for binomial tests for 10 different threshold values. The extrinsic omission rate represented the fraction of the test localities that were assigned into pixels which are not predicted as suitable for Medfly. Low omission rate is highly advisable for a good model [179].

## Results

### Occurrence data

The search for data on Medfly historical occurrence resulted in records from 43 countries and nearly 500 unique localities (Dataset S1). The oldest records come from 1898 and the most recent from 2011. 171 locations contained information about the year of Medfly occurrence, and 125 about a specific month where Medfly was observed. The majority of the records identified Medfly occurrence at the adult stage of development, with some in the pupae and larvae stage. Some of the most common hosts included apricot, guava, peach, various types of citrus (mainly varieties of orange and mandarine), apple, fig, peach, loquat and coffee. The dominant method of recording occurrence data was through food or Trimedlure baited traps (McPhail and Jackson, but also Nadel, Maxitrap, Steiner and Lynfield) [180].

**Figure 1:**
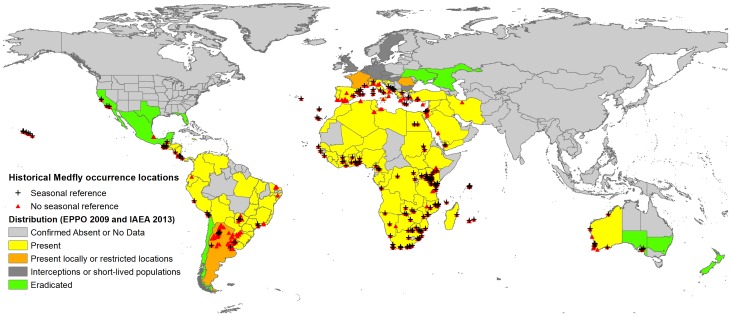
Occurrence data for *Ceratitis capitata* used in the study. Data with information about the month of occurrence is marked with red triangles. Countries/regions where Medfly is present are coloured with yellow and where it is eradicated are marked with green [12], [13].

For the purpose of niche modelling, points with uncertain locations, duplicate points and those collected before 1980 were removed for the analysis. Additionally, the data were supplemented with occurrence points derived from the GBIF, BeBIF and MCA datasets, which further increased the total number of sample points available for niche modelling. In the annual model (produced using all geolocated points available), 463 unique occurrence points were used. For the seasonal models, 139 points were used for the January through April model, 158 for May through August and 157 for September through December. Figure 1 shows the locations of Medfly occurrence worldwide obtained from all of the data sources, starting from 1980 onwards. The points where information about Medfly occurrence after 1980 were available are marked in red (463 unique locations), and those marked with crosses are locations that had the month of record information on file (270 unique locations).

### Environmental niche modelling

**Figure 2:**
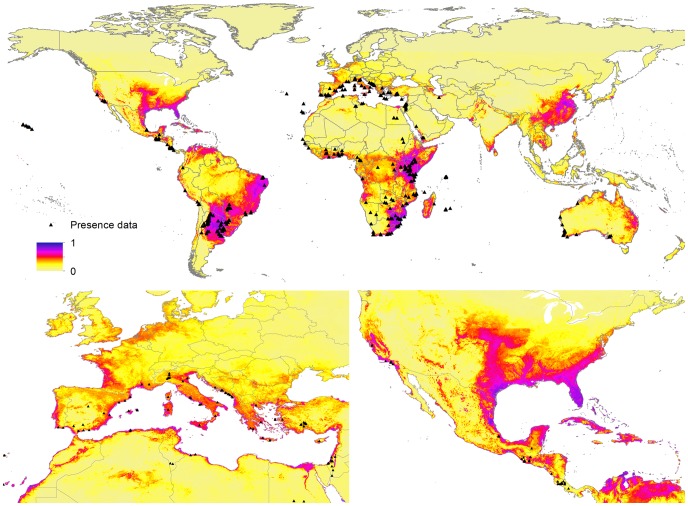
Global environmental suitability for *C. capitata* occurrence as predicted by MaxEnt model. Black triangles represent presence points used in the modeling. Blue, purple and red colors show high confidence in predicted suitability, while yellow represents low confidence and predicted absence.

The annual Medfly niche suitability model, produced using all geolocated occurrence records since 1980, is presented in Figure 2. The largest suitable areas for Medfly presence are located in South America, east and south Africa and eastern Asia. Other suitable areas appear across a variety of climate zones, including warm temperate and semi-tropical and tropical, mostly in coastal areas. This incudes the Mediterranean basin, Gulf of Mexico, western coast of South America and coastal areas of India and Australia. The model prediction performs significantly better than random with a binomial test result of *p*<3.9^−40^. The AUC score for the training and testing datasets is 0.882 and 0.878 respectively, representing strong predictive performance, given the fractional predicted area of 0.307 (Table 3).

**Table 3:** Models were calibrated using training and test data (75% and 25% randomly selected occurrence points respectively).

Model	General	Jan–Apr	May–Aug	Sep–Dec
No. of points	463	139	158	157
Mean training AUC	0.882	0.891	0.866	0.881
Mean test AUC	0.878	0.855	0.848	0.853
Test AUC standard deviation	0.012	0.025	0.025	0.022
Mean fractional predicted area	0.307	0.376	0.335	0.336
Training omission rate	0.097	0.098	0.098	0.098
Test omission rate	0.118	0.165	0.130	0.161
*p* value	3.948^−40^	2.495^−9^	3.795^−10^	1.154^−8^

Area under the curve (AUC) was calculated as an average of 30 model replicate runs using subsample run type. Mean AUC and omission rate values were calculated both for test and training data. The mean omission rates are calculated at an arbitrarily chosen cumulative threshold of 10. All model omission results performed significantly better than random (*p*<0.0001).

**Figure 3:**
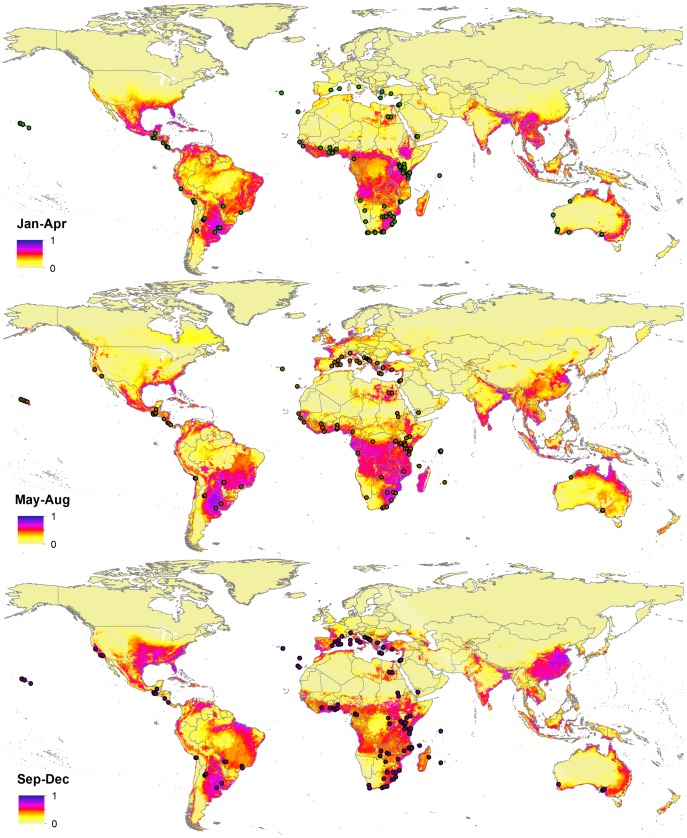
3-panel seasonal maps showing the environmental suitability for *C. capitata* occurrence annually according to the MaxEnt model. Dots represent the seasonal presence points used in the seasonal potential niche modelling. Blue, purple and red colors show high confidence in predicted suitability, while yellow represents low confidence and predicted absence.

The January-April model has the highest fractional predicted area (0.376 for Jan–Apr, 0.335 for May–Aug, and 0.336 for Sept–Dec) (Figure 3). In that season, the least amount of land area in the Mediterranean basin is shown as suitable for Medfly. The area surrounding the Gulf of Mexico and Caribbean Basin, as well as the Pampa in Argentina and eastern Brazil are predicted to be highly suitable. In Africa, the highest suitability is observed in the Sahel belt, some parts of Abisynia and the southern part of the continent, including Madagascar. High suitability is also apparent in southeast Asia, where Medfly is not yet known to be established. The May–Aug season largely corresponds with summer in the northern hemisphere. Consequently, the largest proportions of areas in Europe, North America and Asia appear as suitable, compared to the other seasons. The expansion in predicted suitable range is also apparent in central Africa and northern Australia. In the Sept–Dec season, the suitability is largely contained in the Mediterranean basin in Europe, Southeast United States, but is expanded in the southern hemisphere. The AUC scores for the training data for all the seasons consistently exceed 0.86, while for the test data it remains above 0.84 (Figure S1, Table 3). As in the case of annual suitability model, the seasonal predictions return extremely low *p* values, indicating that the models perform significantly better than random (Table 3).

### Relative importance of predictor variables

Table 4 represents the relative influence of the variables on the model. The most significant environmental contributor appears to be temperature (minimum for the general model and Jan-Apr season and average for the Sep–Dec season). In May–Aug maximum precipitation appears as the most important predictor. These are followed by NDVI, DEM and minimum precipitation.

**Table 4:** Relative influence of the contribution of the variables to the model [%].

Variable	General	Jan–Apr	May–Aug	Sep–Dec
Land Surface Temp (LST) minimum	63.7	32.5	-	3.4
LST maximum	23.3	5.8	-	-
Elevation (DEM)	7.4	7.9	10.2	6.1
Precipitation maximum	2.8	5.9	24.7	14.5
Normalized Difference Vegetation Index (NDVI)	2.0	8.4	18.1	8.8
Precipitation minimum	0.8	9.1	3.5	1.1
LST average	-	-	4.2	17.4
General Model suitability index >0.1 mask	-	30.4	39.3	48.6

## Discussion

The distribution model outputs represent the first global assessment of the seasonally changing potential distribution of Medfly, illustrating the significant shifts in environmental suitability that occurs throughout a typical year. Previous mapping has rarely addressed these seasonal variations, and the output maps provide a basis for global assessments of shifting invasion risks. Some areas identified in this study as highly suitable do not have Medfly populations established at present and this may be a result of either lack of introduction, eradication efforts or presence of another dominant species, such as in the case of eastern Australia, where the Queensland fruit fly has displaced Medfly [66]. The seasonal prediction maps reflect changes in the environmental suitability for Medfly, and it should be noted that while the insect may be able survive in the regions shown to be suitable for one or two of the three seasons mapped, it may likely not be able to become established, due to unsuitable conditions for the remainder of the year. In the locations close to the northern or southern boundary of Medfly distribution, the insect may be able to survive for one or several unusually warm seasons, and not be able to establish a stable population long-term.

The seasonal environmental niche mapping can be an important strategic tool for tailoring control and surveillance activities. With sufficient amounts of spatio-temporal data, the times of year when the pest is at its lowest activity stage can be identified and combined with information about commodity and passenger movements. It can facilitate prioritization and optimization of border surveillance efforts that are operating under limited resources and staffing. It can also be used to target interventions, enable or deny seasonal trade and be incorporated into risk assessments for commodity importation. This methodology, in principle, has the potential to be applied to any invasive insect species, or any organism subject to seasonal population dynamics and density. Combined with information about changes in seasonal movement of commodities at risk or passenger luggage, it could be adopted widely, both in the scientific and policy-making communities.

Our global annual suitability model presented here can be compared with previous published studies on Medfly range. One of the most recent ones was performed using two approaches: a genetic algorithm for rule-set prediction (GARP) and principal component analysis (PCA) [16]. A comprehensive native and non-native dataset on Medfly occurrence was compiled and used in modeling, together with eight environmental covariates consisting of temperature and precipitation parameters. In the model output areas of “high” and “low” suitability indicated by various shades of gray were presented, but thresholds for the division between them were not specified. The GARP model was judged to perform better by the authors; therefore we compare our results to the GARP output. It is noticeable that the MaxEnt annual model presented here tends to be more conservative and return a narrower range of Medfly suitability. It is particularly apparent in Africa and Australia. The models show less agreement in terms of suitability in Americas and good agreement on the suitability in Europe and Asia. Another previous study used CLIMEX to predict Medfly's suitable niche [17]. This model inferred the climatic conditions it can tolerate, based on the CLIMEX Growth Index. Additional modeling was then performed incorporating the effect of irrigation on Medfly abundance. Suitability was illustrated by 3 different suitability indices represented by various sized dots. In this case, the MaxEnt annual model presented here shows substantially closer agreement in terms of the most suitable areas in both the Americas and Europe, while it shows a more constrained suitability range in Africa, Australia and Asia. The study by Gutierrez and Ponti [19] represents a mechanistic approach to Medfly suitability range prediction. They developed a fine scale temperature driven and physiologically-based demographic model for Medfly in order to predict potential distribution in California, Arizona and Italy under the most recent climatic conditions for several individual years and under hypothetical climate warming. Results suggested that the climate in Arizona is outside of the climatic envelope for Medfly, whereas most of the Central Valley of California has marginal suitability, except in south coastal California. They conclude that continuous inter-annual suitability for Medfly could occur only in south coastal regions of the state. Our general Medfly suitability prediction defines a larger portion of California as being suitable – including coastal areas and the Central Valley, while the seasonal model outlined here defines the most suitable conditions as being in the third trimester. During the first trimester high altitude areas are defined as unsuitable, most likely due to low temperature and the second trimester defines most of California as moderately suitable. Stable suitability for Medfly is predicted in the southern part of the Italian peninsula, along the coast near Rome and on the plain of the Po River. Our model shows good agreement with the Gutierrez and Ponti [19] predictions here. Another MaxEnt prediction for Medfly was carried in the past, with default software settings with the exception of increased numbers of iterations. A smaller number of occurrence points was used and a wide range of climate predictors related to temperature and precipitation, without excluding those with high pairwise correlation values, was applied [20]. The model showed the highest suitability for Medfly in the southern part of China, whereas our model depicted another territory of potential invasion in the eastern part of the country.

The database constructed here includes a new level of detail regarding Medfly occurrence, including the sampling method, host type and relative abundance of Medfly in different months. In terms of data coverage, Medfly occurrence is relatively well documented in Mediterranean Europe, but most data from the Middle East comes from Israel, with little information available from other countries in the region. While there do exist comprehensive data on the native range of Medfly across most of Africa, we found few records from the northern part of the continent. Data collection for Central and South America resulted in generally sparse coverage, with Argentina being an exception, where many comprehensive studies were performed and a large amount of occurrence data are available. Only around 25% of records gathered included information about the month of Medfly occurrence. We have received information on Medfly detection locations for Hawaii and California, and no geo-referenced locations for Florida. Given the environmental sensitivity of the species and the resultant significant seasonality in distributions and abundance, future studies should ideally prioritize the collection and assembly of such valuable temporal information.

The seasonal suitability maps presented in this paper show considerably lower suitability for Medfly activity in the northern fringes of its distribution in the first 4 months of the year, which is in agreement with both gathered occurrence data, and previous studies on its seasonal dynamics in the region [3], [18]. During these months Medfly tends to overwinter as larvae in host fruits or at certain extension in a pupal stage in the ground. The adult activity is not present or is very limited. Most of the Mediterranean basin appears as unsuitable and significantly lower suitability is observed in California, where there is discussion about the pest's ability to overwinter. In May–Aug period, the suitability for Medfly extends well above its northern distribution limit in Europe, and a significantly higher suitability is observed in the Mediterranean basin. The highest suitability is observed in the last season, which corresponds well to previous studies on pest seasonal dynamics in the northern hemisphere. Lesser variability in the suitability for Medfly development is observed on the southern fringes on its distribution – especially in South America. More pronounced variability is observed in Australia, mostly in northern parts of the country, which lie outside of the current pest distribution. Few studies on the seasonal activity of Medfly outside of Europe exist and therefore it is hard to evaluate the results with the detection data. More pronounced variability in suitability is observed in near-equator or tropical locations, which might be a result of variability in precipitation and its effect on pupal development, and also host availability and phenology.

Significant uncertainties in the outputs presented here remain. The models have been built on the most comprehensive dataset of Medfly occurrence points yet assembled, but this still has a limited amount of data in many parts of the world. Moreover, the data are often lacking consistency in sampling methodology and possibly subject to errors in spatial and temporal referencing that are difficult to track. Further, while a detailed set of global seasonal environmental covariate datasets has been assembled and utilized in the modeling here, some additional factors could be taken into account in future studies, including humidity. Additionally, there are many factors that influence Medfly presence and abundance for which global spatial data do not exist – these include, for example, the distribution of competitor species, the distribution of host plants, control method coverage and produce movement patterns (natural and by fruit trade). Spatial data on these would likely improve modeling output fidelity and tackle some of the unexplained variance seen. Finer scale regional approaches might shed more light on local pest dynamics. Despite these caveats, the output maps represent the first attempt to model the global seasonal environmental suitability of one of the World's most economically damaging pest species.

Due to continuous efforts towards its elimination in many countries and trade and custom regulations that aim at reducing the risk of its importation, the presence of Medfly is not necessarily continuous across a region, but fragmented. To get a better picture of the seasonal aspects of Medfly activity, the outputs presented here need to be matched and adjusted to known Medfly suitability areas, where the pest could overwinter and become established. Future work will aim to tackle this and link the seasonal distribution maps presented here with seasonally changing commodity movement and human travel data to work towards building predictive models of Medfly importation risk and how it likely changes seasonally. The analyses presented here have shown how the suitability for Medfly changes throughout a typical year, but the riskiest movements of people and commodities for Medfly importation to suitable areas also change seasonally, and assessing reliably the risk of both Medfly importation and establishment should account for both. Such approaches can likely aid surveillance planning in prioritizing limited resources.

## Conclusions

Very few studies exist on the seasonal modeling of species distributions, and these studies are usually performed on local or regional scales. For species that are highly sensitive to environmental conditions that display strong seasonal patterns in distributions and abundances, seasonal modeling of environmental suitability can be crucial in terms of understanding and predicting when and where a pest is most likely to be at the peak of its population activity, potentially informing targeted surveillance at borders. Continued effort in gathering information about Medfly detection locations, not only in terms of spatial occurrence, but recording activity on seasonal scales, can serve as a tool to understand the spatio-temporal population dynamics of the species. Increasing numbers of tools are available to model species potential distributions, and with finer resolutions of global spatial environmental datasets as well as ever increasing computing power to handle such large datasets more accurate prediction of species potential distributions can likely be performed. Even the most robust methods however, are limited in their performance where occurrence data is incomplete or scarce. Continued efforts to document Medfly and other pest species occurrence and make such records available are therefore essential for improvement of our understanding and prediction of their distributions.

## Supporting Information

Figure S1 **ROC curves.** Red lines represent mean AUC and mean +/− one standard deviation (blue field). The lines show the “fit” of the model to the training and test data. Black line represents random prediction. The red line can be considered to show the real test of the models predictive power [175].
(DOC)

Dataset S1 **Medfly occurrence dataset.**
(XLSX)
