# Supplementary material for: Global Assessment of Seasonal Potential Distribution of Mediterranean Fruit Fly, Ceratitis capitata (Diptera: Tephritidae)
Source: PLoS One. 2014 Nov 6;9(11):e111582. doi: 10.1371/journal.pone.0111582 (PMC4222914; doi:10.1371/journal.pone.0111582)

**Figure S1.** Receiver operating characteristic (**ROC) curves.** Red lines represent mean area under the curve (AUC) and mean +/- one standard deviation (blue field). The lines show the “fit” of the model to the training and test data. Black line represents random prediction. The red line can be considered to show the real test of the models predictive power [38].


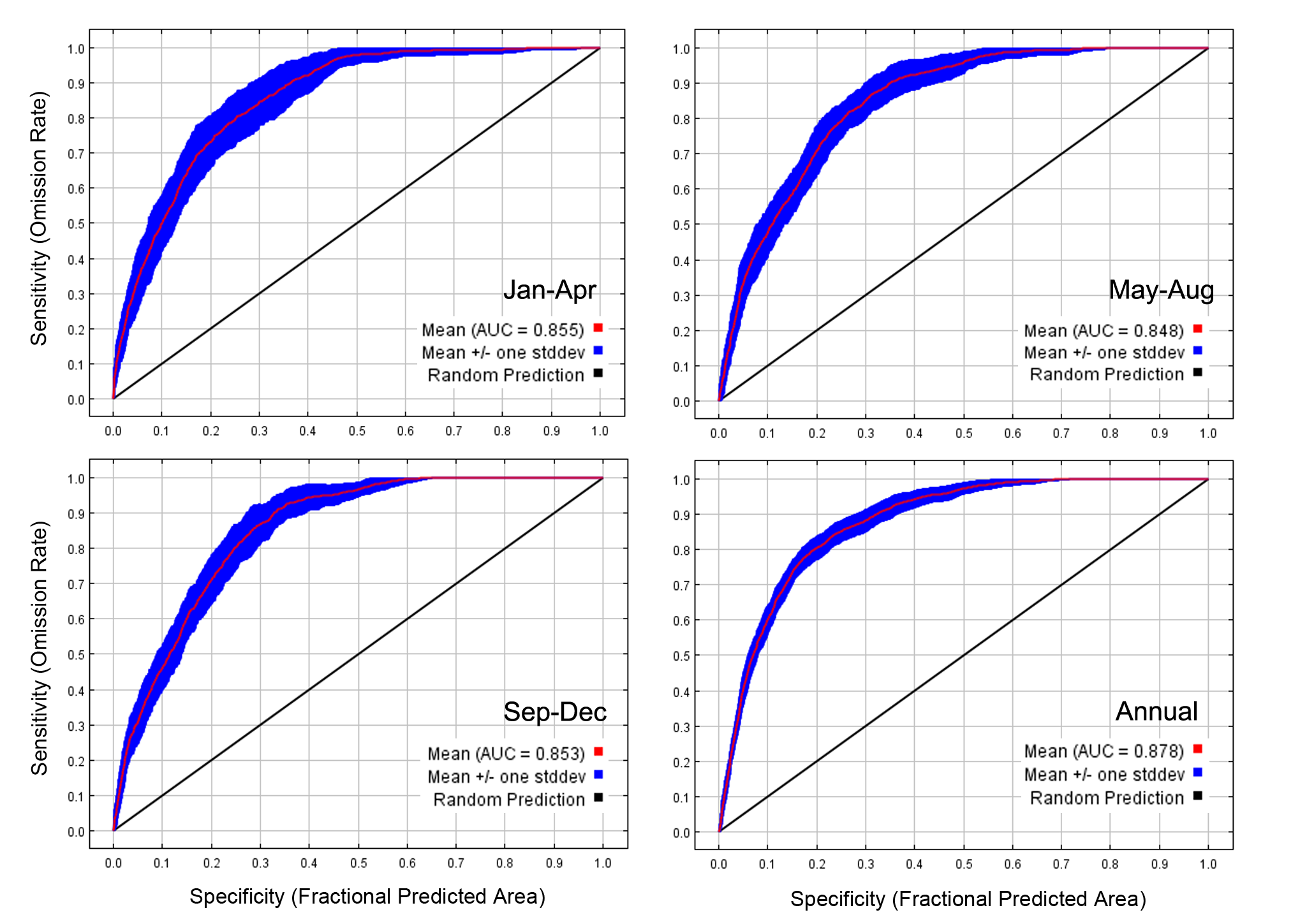

Supplement: Figure S1 — ROC curves. Red lines represent mean AUC and mean +/− one standard deviation (blue field). The lines show the “fit” of the model to the training and test data. Black line represents random prediction. The red line can be considered to show the real test of the models predictive power [175]. (DOC) [file pone.0111582.s001.doc]
